# A Metabolic Stress Response

**DOI:** 10.1093/geroni/igaa057.404

**Published:** 2020-12-16

**Authors:** Scott Leiser, Christopher Choi, Ajay Bhat, Charles Evans

**Affiliations:** 1 University of Michigan, Ann Arbor, Michigan, United States; 2 University of Michigan Medical School, Ann Arbor, Michigan, United States; 3 University of Michigan, ANN aRBOR, Michigan, United States

## Abstract

An organism’s ability to respond to stress is crucial for long-term survival. These stress responses are coordinated by distinct but overlapping pathways, many of which have been found to also regulate longevity in multiple organisms across species. Despite extensive effort, our understanding of these pathways and how they affect aging remains incomplete and thus is a key area of study in Geroscience. Our previous work identified flavin-containing monooxygenase-2 (fmo-2) as a key longevity-promoting gene downstream of at least three longevity promoting pathways, including the hypoxic response, the pentose phosphate pathway, and the dietary restriction pathway. Based on the commonalities of these pathways, we hypothesized that fmo-2, a classically annotated xenobiotic enzyme, might play a key endogenous role in responding to metabolic stress. Our resulting data, using metabolic profiling and further epistatic analysis, both support this hypothesis and link fmo-2’s mechanism to modifications to one-carbon metabolism (OCM), a key intermediate pathway between the nucleotide metabolism, methylation, and transsulfuration pathways. Using mathematical modeling and a novel metabolomics approach, we were able to further identify the likely mechanism of fmo-2-mediated metabolic effects, and connect them to both OCM and downstream components. We propose a model whereby nematode fmo-2 represents a class of enzymes that are able to modify large aspects of metabolism, similar to how transcription factors modify gene expression, and that fmo-2 is a key member of a conserved metabolic stress response.

